# Oral Microbiome: *Streptococcus mutans*/Caries Concordant-Discordant Children

**DOI:** 10.3389/fmicb.2022.782825

**Published:** 2022-02-17

**Authors:** Márcia Dinis, Melissa Agnello, Lujia Cen, Bhumika Shokeen, Xuesong He, Wenyuan Shi, David T. W. Wong, Renate Lux, Nini Chaichanasakul Tran

**Affiliations:** ^1^Section of Pediatric Dentistry, School of Dentistry, University of California, Los Angeles, Los Angeles, CA, United States; ^2^Section of Oral Biology, School of Dentistry, University of California, Los Angeles, Los Angeles, CA, United States; ^3^M2Biome LLC, San Francisco, CA, United States; ^4^Microbiology, The Forsyth Institute, Cambridge, MA, United States; ^5^Section of Periodontics, School of Dentistry, University of California, Los Angeles, Los Angeles, CA, United States; ^6^Center for Oral/Head and Neck Oncology Research, Laboratory of Salivary Diagnostics, School of Dentistry, University of California, Los Angeles, Los Angeles, CA, United States

**Keywords:** caries, *Streptococcus mutans*, saliva, microbiome, pediatric dentistry

## Abstract

Dental caries remains the most common chronic disease in children, and the respective etiology is not fully understood. Though *Streptococcus mutans* is an important factor in the initiation and progression of caries, its presence is not always associated with the disease. The existence of caries discordant populations, in which *S. mutans* counts do not correlate with caries experience, poses a challenging problem. This study explored the possible correlation of S. *mutans* and other microorganism levels on caries-associated ecology of caries-concordant and discordant populations. A total of forty-seven children were analyzed in this study and stratified into four clinical groups based on their *S. mutans* levels in saliva (HS/LS: High/low *S. mutans*) and caries experience. *Streptococcus mutans* levels were determined by culture-based selective plating. The salivary microbiome of caries concordant and discordant populations was investigated by 16S rRNA gene sequencing and downstream bioinformatics analysis. The salivary microbial communities significantly clustered based on *S. mutans* levels and independent of their caries experience. In addition to *S. mutans* levels, significant differences in the abundance of other species were observed between HS and LS groups. Interestingly, disease-associated species such as *Veillonella dispar, Streptococcus* spp., and *Prevotella* spp. were significantly increased in HS groups and may contribute, in combination with *S. mutans*, to the caries progression. Furthermore, health-associated species exhibited higher abundance in the LS groups, such as *Veillonella rogosae, Haemophilus* sp., and *Alloprevotella* spp. but their possible contribution to the caries process remains to be elucidated. This study provides evidence that *S. mutans* may play a role in shaping the salivary microbial community. Our results highlight that future caries research should consider additional species as health/disease microbial markers in conjunction with *S. mutans* to improve diagnosis and caries management of the caries-discordant population.

## Introduction

Dental caries is one of the most prevalent childhood diseases in the United States and worldwide (Dye et al., 2015; Kassebaum et al., 2017). Although considered a preventable disease, the persistence of high caries necessitates the improvement in diagnostics and therapeutic interventions for young and vulnerable populations. A multifactorial etiology of dental caries comprises of a complex interaction of acid-producing bacteria, fermentable carbohydrates, and host factors (Selwitz et al., 2007).

From the microbiological perspective, *Streptococcus mutans* has been considered a key pathogen in the initiation and progression of caries (Tanzer et al., 2001). Hence, *S. mutans* levels have been used as part of caries risk assessment (Guo and Shi, 2013; Edelstein et al., 2016). However, contradicting reports exist in the literature regarding the correlation between *S. mutans* counts and caries experience. Previous studies established a causal relationship between *S. mutans* levels in plaque or saliva and caries experience in children (Alaluusua and Renkonen, 1983; Loesche, 1986; Damle et al., 2016; Edelstein et al., 2016), while others reported distinct populations whose caries status is not correlated with *S. mutans* levels have been observed (Carlsson et al., 1985; Matee et al., 1992, 1993). Furthermore, studies in subjects with rampant caries did not reveal detectable *S. mutans* levels (Aas et al., 2008). These seemingly contradicting results raised awareness of previously untapped interesting caries discordant clinical population: caries-free children with high *S. mutans* levels, as well as those with low *S. mutans* levels who are severely affected by dental caries.

One possible explanation for the existence of discordant populations could be the ecological plaque hypothesis (Marsh, 1994), which echoes the fact that *S. mutans* may not be the sole etiological factor. In this scenario, caries would result from a disruption of the homeostasis of the resident microflora driven by changes in local environmental conditions (Wade, 2013). Emerging evidence associated additional bacterial species within the salivary microbiome with caries experience in children. Besides *S. mutans*, other acid-producing microorganisms such as certain species of the genera *Veillonella*, *Scardovia*, and *Lactobacillus* have also been identified in caries-affected children populations (Guo and Shi, 2013; Jiang et al., 2016; Xu et al., 2018). Additionally, there are studies linking *Actinomyces* and *Bifidobacterium* genera with cavitated caries lesions (Brailsford et al., 1999; Aas et al., 2008; Mantzourani et al., 2009; Guo and Shi, 2013). Despite a considerable body of evidence, the microbiological ecology of the caries etiology is not fully understood, limiting our ability to explain the existence of the caries discordant population. Moreover, a comprehensive investigation of the oral microbiome in this population is scarce.

In this study, we investigated the caries concordant-discordant clinical populations for their salivary *S. mutans* levels and the respective microbiome. These unique clinical populations may hold the key to a better understanding of the ecological relationship between *S. mutans* and other microbial species and the impact on dental caries in children.

## Materials and Methods

### Ethics

The study was reviewed and approved by the University of California, Los Angeles Institutional Review Board (13-001075). Informed written consent was obtained from parents or legal guardians of all participants prior to initiation of the study. Additionally, child assent was obtained from participants that were considered to be capable of providing assent, taking into account their age, maturity, and psychological state.

### Recruitment and Sampling

Study participants were recruited from the pediatric patient population of the Children’s Dental Center at the University of California, Los Angeles (UCLA). A total of sixty healthy (ASA I) children between 4 and 14 years of age were recruited and enrolled in the study. Each participant completed a brief questionnaire on demographics, oral hygiene habits, and dental treatment history. Participants were enrolled based on the following inclusion criteria: children, who were not taking any medication, and had no antibiotic usage within the past 6 months. Participants were excluded from the study if they had generalized rampant caries, periodontitis, halitosis, open sores or ulcerations, chronic systemic diseases, reduced saliva production. Oral clinical evaluation and radiographic exams were performed by a single pediatric dental resident at the UCLA School of Dentistry. The caries experience was evaluated using the decayed, missing, and filled tooth (dmft/DMFT) index, according to the criteria proposed by World Health Organization (1997).

All participants were asked to refrain from oral hygiene procedures, eating and drinking for at least 2 h before oral sampling. Saliva samples were collected between 8:00 am and 10:00 am. Participants were instructed to rinse their mouths with water prior to collection. Then, 5 mL of unstimulated saliva was collected by drooling/spitting directly into collection tubes or through direct sampling using a soft, sterile plastic pipette (Henson and Wong, 2010). The collected sample was split, one aliquot was immediately used for *S. mutans* levels quantification via plating on selective media, and the remaining portion was stored in 20% glycerol at −80°C until further analysis.

The participants were stratified into four groups based on their *S. mutans* levels and caries experience: high (HS, ≥1.0 × 10^5^ CFUs/mL) and low (LS, <1.0 × 10^4^ CFUs/mL) *S. mutans* counts; and high (HC, dmft/DMFT ≥ 4) and low (LC, dmft/DMFT < 2) caries scores. Of the samples derived from the initially enrolled sixty children, forty-seven passed additional quality control for microbiome sequencing and were subsequently included in the study: HSHC (high *S. mutans*/high caries, *n* = 13), HSLC (high *S. mutans*/low caries, *n* = 10), LSHC (low *S. mutans*/high caries, *n* = 13), and LSLC (low *S. mutans*/low caries, *n* = 11) (Table 1).

**TABLE 1:** Demographic and oral hygiene information from the clinical study questionnaire.

Characteristic	HSHC (*n* = 13)	HSLC (*n* = 10)	LSHC (*n* = 13)	LSLC (*n* = 11)
**Gender**				
Female	10	6	3	7
Male	3	4	10	4
**Ethnicity**				
Hispanic	10	10	12	5
Non-Hispanic	3	0	1	6
**Age**	(4–12 years)	(4–14 years)	(4–12 years)	(4–13 years)
	**7.7 ± 2.5**	**10 ± 3.0**	**8.3 ± 2.1**	**9.2 ± 2.9**
**dmft/DMFT index**	(4–10)	(0–2)	(4–10)	(0–2)
	**6.5 ± 2.3**	**1.1 ± 0.9**	**4.8 ± 1.5**	**0.2 ± 0.6**
**Professional dental cleaning frequency**				
None	0	2	3	0
Once a year	2	1	0	2
More than once a year	11	7	10	9
**Tooth brushing frequency**				
Not brushing	0	0	0	0
Once a day	3	0	0	1
More than once per day	10	10	13	10
**Tooth Flossing frequency**				
No flossing	2	3	5	1
Less than once a day	0	0	0	0
Once a day	8	4	1	1
More than once per day	3	3	7	9

*High S. mutans: ≥1.0 × 10^5^ CFU’s/mL.*

*Low S. mutans: <1.0 × 10^4^ CFU’s/mL.*

*High Caries: dmft/DMFT ≥ 4.*

*Low Caries: dmft/DMFT < 2.*

*HSHC, High S. mutans High Caries; HSLC, High S. mutans Low Caries; LSHC, Low S. mutans High Caries; LSLC, Low S. mutans Low Caries.*

*The bold values correspond to the average of age and caries experience parameters per group and their respective standard deviations.*

### Quantification of Salivary *S. mutans* Levels

Saliva samples were serially diluted, and 100 μl of each dilution was plated on brain heart infusion (BHI, Bacto™ Brain Hearth Infusion, Becton, Franklin Lakes, NJ, United States, Dickinson and Company, Franklin Lakes, NJ, United States) and selective mitis-salivarius-bacitracin (MSB, Difco™ Mitis Salivarius Agar, Becton, Franklin Lakes, NJ, United States, Dickinson and Company, Franklin Lakes, NJ, United States) agar plates. The plates were incubated anaerobically at 37°C for 2 days. Total bacteria were counted on BHI plates, while *S. mutans* was identified and enumerated according to their specific colony morphology on the MSB selective plates. Colonies were further verified by an *S. mutans*-specific monoclonal antibody as previously described (Gu et al., 2006). Briefly, 10 μL of collected sample was mixed with 10 μL of hybridoma cell line culture supernatant producing an anti-*S. mutans* moboclonal antiboby (Mab) (10 μg/μL); incubated at room temperature for 30 min. Then, 1 μL of fluorescein isothiocyanate (FITC)-conjugated goat anti-mouse immunoglobulin G (IgG) was added and incubated at room temperature for 30 min. The mixture was examined using fluorescent microscopy.

### DNA Extraction

Total genomic DNA was extracted from the saliva samples using the Epicenter MasterPure™ DNA purification kit (Lucigen, United States), following the manufacturer’s instructions with modifications. Briefly, saliva samples were subjected to mechanical grinding with glass beads (425–600 μm, Sigma), for five cycles of 1-min gridding and 1-min stationary, followed by lysozyme treatment for 2 h at 37°C (Agnello et al., 2017). The DNA quantity and quality were measured with NanoDrop 2000 (Thermo Fisher Scientific, Waltham, MA, United States). The extracted DNA was stored at −20°C until further use.

### Salivary Microbial Community Analysis

The DNA extracted from the saliva samples was sent for microbiome sequencing at the UCLA Microbiome Core. Amplicon libraries were prepared in triplicate by using 16S Metagenomic Sequencing Library Preparation Preparing 16S Ribosomal RNA Gene Amplicons for the Illumina MiSeq System, Illumina ^®^ according to the manufacturer’s instructions. Briefly, 10–50 ng of genomic DNA was used as a template in a PCR reaction to amplify the V4 region of the 16S rRNA encoding gene and barcoded using 515f/806r primers. Afterward, the product was purified using AMPure beads (Beckman Coulter) and hundred ng of each library was pooled, gel-purified, and quantified (Bioanalyzer, Agilent), and 12 pM of the mixture, spiked with 20% PhiX, and sequencing was performed on an Illumina MiSeq sequencer system (Illumina, San Diego, CA, United States).

Sequencing reads were de-multiplexed and adaptor sequences removed. Quality filtering removed bad reads and chimeric sequences prior to analysis. Sequencing data were analyzed using Quantitative Insights into Microbial Ecology (QIIME) version 1.9.1 (Caporaso et al., 2010). Sequences were clustered into operational taxonomic units (OTUs) using UCLUST, aligned, and the taxonomy was assigned with the Human Oral Microbiome Database (HOMD) as reference. For determination of alpha diversity, OTU tables were rarefied to 93,000 reads, and Shannon indices were calculated. For the analysis of beta diversity, weighted UniFrac distances were calculated, followed by principal coordinates analysis (PCoA).

### Statistical Analysis

The statistical tests were performed using GraphPad Prism (GraphPad Prism version 8.0.0, GraphPad Software, San Diego, CA, United States), and statistical significance was defined as follows: *p < 0.05, ^**^p < 0.005, and ^***^p < 0.0005.

The demographic and *S. mutans* quantification data were compared between groups and assessed for the significance of their differences with one-way ANOVA followed by Kruskal–Wallis multiple comparisons test. Spearman correlation analysis was performed to investigate possible correlations between demographic and oral hygiene parameters.

For salivary microbiome sequencing data, differences in the relative abundances of taxa between the groups were determined with the Kruskal–Wallis test, controlling the false discovery rate to correct for multiple comparisons, and a corrected *p* ≤ 0.05 was considered significant. Differences in weighted UniFrac distances between the groups were analyzed with analysis of similarity (ANOSIM), and *p* ≤ 0.05 was considered statistically significant.

## Results

### Study Demographics

A total of forty-seven children were enrolled and stratified into four groups based on their *S. mutans* levels and caries experience: HSHC (*n* = 13), HSLC (*n* = 10), LSHC (*n* = 13), and LSLC (*n* = 11). The respective age, gender, ethnicity, dmft/DMFT index, and oral hygiene parameters of the participants are summarized in Table 1. In brief, the gender distribution was 26 females (55.3%) and 21 males (44.7%), and their ages ranged from 4 to 14 years with an average of 8.8 (±2.7) years. The study population was comprised of 78.7% Hispanic and 21.3% non-Hispanic children, and the caries experience varied from a dmft/DMFT score of 0 to 10, with an average of 5.6 (±2.1) in the high caries groups (HSHC, LSHC) and 0.6 (±1.0) in the low caries groups (HSLC, LSLC). The frequency of their oral hygiene habits, such as professional cleaning, as well as their tooth brushing, mouth rinse, and flossing routine, did not vary between groups. Overall, there were no significant differences between the groups for all of the above-mentioned parameters, except for the caries status that was a significant difference between high and low caries groups.

### Caries Experience and *Streptococcus mutans* Levels in Saliva

**FIGURE 1:**
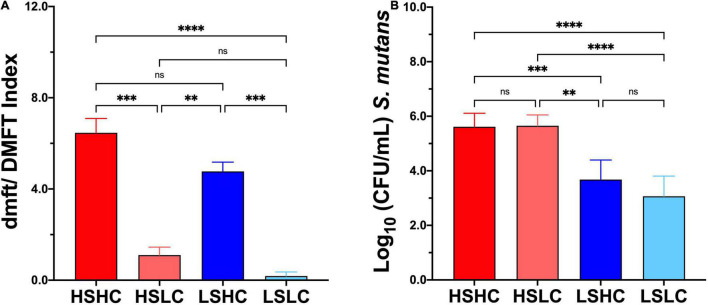
Caries experience and salivary *Streptococcus mutans* levels quantification. **(A)** Caries status was assessed using decayed, missing, and filled tooth (dmft/DMFT) criteria, and the graphic represents the average caries experience. **(B)** *S. mutans* levels were evaluated by selective culture media method, and the graphic represents the mean of *S. mutans* Colony Forming Units (CFU/mL), per group (HSHC, HSLC, LSHC, and LSLC), and error bars represent the standard deviation (SD). Differences in significance between groups were analyzed using one-way ANOVA followed by Kruskal–Wallis multiple comparisons tests; ^**^p < 0.005, ^***^p < 0.0005, ^*⁣*⁣**^p < 0.00005.

While the caries status for both HC (HSHC, LSHC) and LC (HSLC, LSLC) groups was similar to each other, differences in dmft/DMFT between groups reflecting the contrasting caries experience were statistically significant (HSHC vs. HSLC, HSHC vs. LSLC, HSLC vs. LSHC, and LSHC vs. LSLC) (Figure 1A). For salivary *S. mutans* quantification, as expected, there were no significant differences between groups with similar *S. mutans* levels (HSHC vs. HSLC, and LSHC vs. LSLC) despite the difference in caries experiences (Figure 1B). Only those with dissimilar *S. mutans* levels were statistically different (HSHC vs. LSHC, HSHC vs. LSLC, HSLC vs. LSHC, and HSLC vs. LSLC) (Figure 1B).

### Salivary Microbiome Composition of Clinical Populations

Next, the salivary microbiome of caries concordant and discordant clinical populations was investigated. A total of 8,671,057 high-quality sequences from forty-seven samples, with a mean of 108,490 ± 30,338 (range 93,113–230,591), were generated after processing the data obtained from 16S rRNA amplicon sequencing.

**FIGURE 2:**
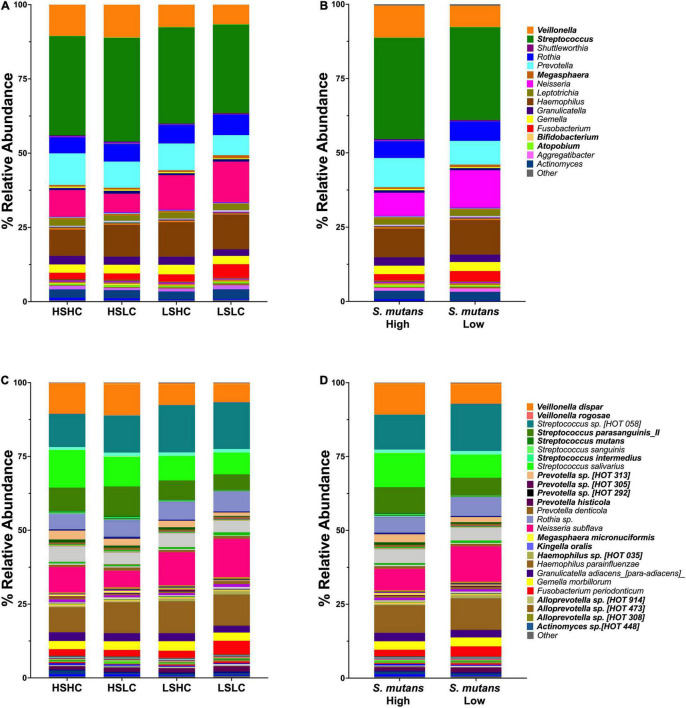
Genus and species level analysis of salivary microbiome community. Relative abundances are shown for salivary microbial profiles at genus **(A,B)** and species **(C,D)** level. **(A,C)** Per group (HSHC, HSLC, LSHC, and LSLC). **(B,D)** *S. mutans* levels (High, Low). Statistical significance was evaluated by one-way ANOVA followed by Kruskal–Wallis multiple comparisons tests.

Taxonomy analysis revealed 11 phyla, of which the Firmicutes was the only phylum that significantly differed between the samples with high and low *S. mutans* levels (55.3 vs. 47.3%, *p* = 0.02). A total of 120 genera comprising 310 species were detected. The salivary microbial community composition based on clinical groups, *S. mutans* levels, and caries experience is represented at the genus (Figures 2A,B) and species levels (Figures 2C,D). Twelve major genera, which constituted 90% of the total, dominated the communities and: *Streptococcus* (32.6% ± 2.1), *Haemophilus* (10.7% ± 1.3), *Neisseria* (10.0% ± 3.3), *Veillonella* (8.9% ± 2.2), *Prevotella* (8.8% ± 1.6), *Rothia* (6.1% ± 0.6), *Gemella* (2.9% ± 0.2), *Fusobacterium* (2.9% ± 1.2), *Actinomyces* (2.9% ± 0.3), *Granulicatella* (2.6% ± 0.3), *Leptotrichia* (2.0% ± 0.2), and *Aggregatibacter* (1.1% ± 0.3). Of these, only the abundance of *Veillonella* was significantly different between groups with high and low *S. mutans* levels (10.7 vs. 7.1%, *p* = 0.003). The abundance of *Streptococcus* appeared elevated in samples with high *S. mutans* levels (34.0 vs. 31.2%), while levels of the genera *Neisseria* (7.7 vs. 12.5%), *Rothia* (5.7 vs. 6.6%), and *Fusobacteria* (2.3 vs. 3.5%) were lower, albeit those differences were not statistically significant. Of note, the genera *Megasphaera*, *Atopobium*, and *Bifidobacterium* were significantly elevated in samples with high *S. mutans* levels (Table 2). Moreover, within the high *S. mutans* groups all these genera were higher in the HSLC compared to the HSHC group.

**TABLE 2:** Relative abundance of the genus and species- level OTUs detected in the saliva, that were significantly different between high and low *S. mutans* samples.

% Relative abundance	*S. mutans* levels		
Genus – level	High	Low	*p*-value
Atopobium	0.360%	0.168%	**0.035**
Bifidobacterium	0.067%	0.001%	**0.009**
Megasphaera	0.485%	0.342%	**0.035**
Veillonella	10.732%	7.098%	**0.031**

**Species – level**	**High**	**Low**	***p*-value**

*Actinomyces* sp. [HOT 448]	0.019%	0.007%	**0.016**
*Alloprevotella* sp. [HOT 473]	0.050%	0.216%	**0.023**
*Alloprevotella* sp. [HOT 914]	0.048%	0.113%	**0.044**
*Haemophilus* sp. [HOT 035]	0.321%	0.966%	**0.046**
*Kingella oralis*	0.013%	0.004%	**0.016**
*Megasphaera micronuciformis*	0.474%	0.342%	**0.033**
*Prevotella histicola*	0.006%	0.002%	**0.012**
*Prevotella* sp. [HOT 292]	0.015%	0.005%	**0.016**
*Prevotella* sp. [HOT 313]	2.667%	1.697%	**0.023**
*Streptococcus intermedius*	0.286%	0.154%	**0.016**
*Streptococcus mutans*	0.393%	0.006%	**0.000**
*Streptococcus parasanguinis_II*	8.755%	6.062%	**0.054**
*Veillonella dispar*	10.183%	6.432%	**0.016**
*Veillonella rogosae*	0.035%	0.056%	**0.045**

*High S. mutans: ≥1.0 × 10^5^ CFU’s/mL.*

*Low S. mutans: <1.0 × 10^5^ CFU’s/mL.*

*The significant P-value of the group is indicated in bold.*

**FIGURE 3:**
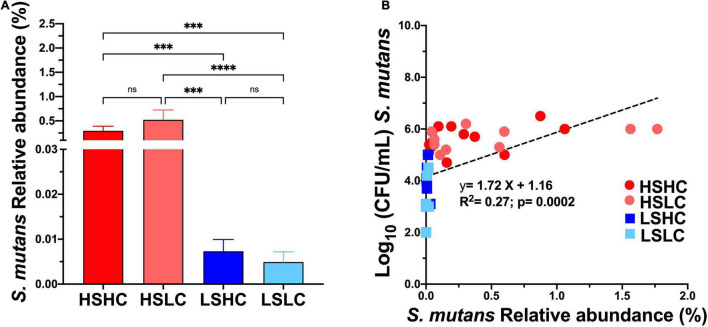
Salivary *S. mutans* levels detected with culture based method versus microbiome sequencing. The relative abundance of *S. mutans* detected on saliva upon 16S rRNA sequencing **(A)** per group (HSHC, HSLC, LSHC, and LSLC). **(B)** Linear regression analysis between the *S. mutans* levels detect by culture based (CFU’s/mL) and 16S RNA sequencing (% Relative abundance). The graphic bars represent means, and error bars represent the standard deviation (SD). Differences in significance between groups were analyzed using one-way ANOVA followed by Kruskal–Wallis multiple comparisons tests; ^***^p < 0.0005, ^*⁣*⁣**^p < 0.00005.

The saliva microbiome analysis at the species level revealed that *S. mutans* abundance was similar within the high (HSHC vs. HSLC, 0.29 vs. 0.52%) and low (LSHC vs. LSLC, 0.007 vs. 0.005%) groups, but significantly different between groups with divergent *S. mutans* levels (HSHC vs. LSHC, HSHC vs. LSLC, HSLC vs. LSHC, and HSLC vs. LSLC) (Figure 3A). Importantly, linear regression analysis demonstrated that the *S. mutans* levels detected by microbiome sequencing were correlated with those quantified using selective culture (Figure 3B).

The correlation between *S. mutans* levels and the abundance of other bacterial species was further analyzed. Fourteen species-level operational taxonomic units were significantly different in the samples with high *S. mutans* levels compared to those with lower levels (Table 2). Samples of the high *S. mutans* group also contained significantly elevated levels of *Veillonella dispar*, *Streptococcus parasanguinis_II*, *Prevotella* sp. [HOT 313], *Megasphaera micronuciformis*, *Streptococcus intermedius*, *Actinomyces* sp. [HOT 448], *Prevotella* sp. [HOT 292], *Kingella oralis*, and *Prevotella histicola*. In the samples with low *S. mutans* levels, significantly higher abundance was observed for *Haemophilus* sp. [HOT 035], *Alloprevotella* sp. ([HOT 473] and [HOT 914]), and *Veillonella rogosae* (Table 2).

**FIGURE 4:**
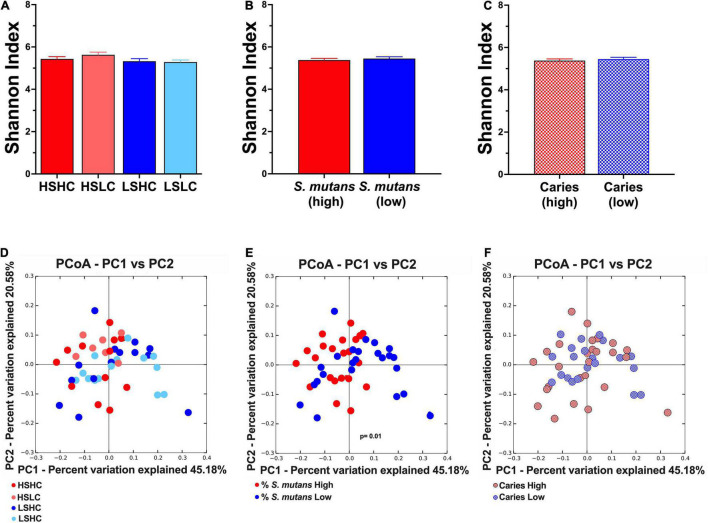
Alpha- and Beta- diversity analyses of salivary microbial community. Alpha diversity was analyzed using Shannon index (upper panel, **A–C**). The beta diversity was by principal coordinates analysis (PCoA) of weighted UniFrac distances (lower panel, **D–F**). The analyses were performed based on the following parameters: **(A,D)** per group (HSHC, HSLC, LSHC, and LSLC); **(B,E)** *S. mutans* levels (High, Low); and **(C,F)** caries experience (High, Low). The graphic bars represent means, and error bars represent the standard deviation (SD). The differences in significance of the groups was performed by one-way ANOVA followed by Kruskal–Wallis multiple comparisons tests (alpha diversity) or ANOSIM (beta diversity).

Furthermore, the analysis of the salivary microbial community diversity revealed that there were no significant differences neither between the analyzed clinical groups (Figure 4A), *S. mutans* levels (Figure 4B), nor the individual caries status (Figure 4C). The beta diversity of the salivary microbial communities was evaluated using weighted UniFrac, and plotted using principal coordinates analysis (Figures 4D–F). The samples were analyzed according to a combination of both *S. mutans* levels and caries experience (HSHC, HSLC, LSHC, and LSLC) as well as the individual parameters. The samples significantly clustered according to their *S. mutans* levels (Figure 4E). Moreover, the samples displayed an apparent pattern associated with their caries status, but this was not statistically significant (Figure 4F).

## Discussion

Dental caries is a multifactorial oral disease affecting children worldwide, for which the complete pathogenesis is not yet fully understood. The existence of caries discordant populations, in which *S. mutans* levels do not correlate with the observed caries experience, poses a compelling challenge for caries management. In this study, we use saliva as a source of *S. mutans* and oral microbiome isolation because it reflects the composition of the oral microbiome, mirrors the complex microbial ecosystems of the oral cavity (Belstrøm, 2020), and is an excellent non-invasive diagnostic biofluid. During the initial phase of random participant recruitment for this study, we observed that only 60% of the clinical population could be categorized into caries concordant groups (HSHC and LSLC). In contrast, a substantial portion of the population (40%) displayed caries discordant phenotypes (HSLC and LSHC), implying that the presence and quantity of *S. mutans* may not be the sole determinant of disease risk. In this study, the possible role of *S. mutans* levels and/or other microorganisms on caries-associated ecology was explored in concordant and discordant populations.

Our results indicate that *S. mutans* levels may play a role in shaping the community as significant differences in the groups with high and low counts of this cariogenic pathogen were observed. In the HS groups, apart from *S. mutans* several other genera and species were significantly higher than in the LS groups (Table 1). The genera with significant differences included the acid producers, *Bifidobacterium* (Henne et al., 2015) and *Atopobium* (Kolenbrander et al., 2009), the lactic acid consumer *Veillonella* (Mashima and Nakazawa, 2014), and *Megasphaera*, which is closely related to *Veillonella* (Nallabelli et al., 2016). Previously, these genera have been identified in close association with high *S. mutans* levels (Aas et al., 2008) that they are thought to either assist in the production or metabolism of lactic acid. Of note *Bifidobacterium dentium* was found to be present in 30.8% of caries active individual but was not present at all in caries-free individual (Henne et al., 2015), suggesting that this species should be considered alongside *S. mutans* as another aciduric taxa contributing to dental caries. Although several studies have recognized the contribution of the genus *Veillonella* in dental caries (Becker et al., 2002; Aas et al., 2008; Gross et al., 2012; Jiang et al., 2016; Agnello et al., 2017; Xu et al., 2018; Hurley et al., 2019), differential roles may exist at species level (Do et al., 2015). In this study, the relative abundance of two different *Veillonella* species were significantly different between groups. While high levels of *V. dispar* were detected in the HS groups, *V. rogosae* was significantly higher in LS groups. Similar to our findings, previous studies have reported an increased abundance of *V. dispar* in caries-active children (Arif et al., 2008; Hurley et al., 2019) and *V. rogosae* in caries-free children (Arif et al., 2008). There is little doubt that the ability of *Veillonella* to consume lactate can contribute to caries development. Interestingly, *M. micronuciformis*, a low abundance species which was significantly increased in the HS groups were previously reported in association with caries (Kalpana et al., 2020).

Other pertinent bacterial species were observed in high abundance in the HS groups. For *Streptococcus* species, besides *S. mutans*, *S. parasanguinis*, and *S. intermedius* were significantly higher in the HS groups. However, their association with health or disease is controversial as elevated levels of these species were reported in both caries-active and caries-free children (Becker et al., 2002; Corby et al., 2005; Aas et al., 2008). In addition, significantly higher levels of caries-associated species such as *Actinomyces* sp. and *Prevotella* spp. were also observed in the HS groups, both of which have been reported in both the saliva and plaque of children with severe caries lesions (Becker et al., 2002; Corby et al., 2005; Jiang et al., 2013, 2016; Ma et al., 2015; Zhang et al., 2015; Zhu et al., 2018; Kalpana et al., 2020). Particularly, a higher abundance of *Prevotella* spp. was correlated with the group characterized by elevated *S. mutans* and caries experience levels. A previous study investigating the microbiome of dentin carious lesions reported similar results (Hurley et al., 2019).

Focusing on the LS groups, no significant trends were observed at the genus level. Besides the health-related *V. rogosae* mentioned above, at the species level, *Alloprevotella* spp. and *Haemophilus* sp. were also significantly increased in the LSLC group. While our findings are consistent with previous reports (Hurley et al., 2019), other studies suggested an association of these species with caries-affected children (Xu et al., 2018; Zhu et al., 2018), yet another group published no distinct association with either one (Jiang et al., 2016).

The microbial community diversity in both the caries concordant and discordant populations were compared based on clinical groups (Figures 4A,D), *S. mutans* levels (Figures 4B,E), and caries experience (Figures 4C,F). Overall, when comparing between clinical groups or caries experience, there were no significant differences in species abundance, richness, diversity, and no particular clustering pattern was observed. Previous studies that applied the same alpha-diversity index only found a correlation with caries experience when the disease severity progressed into dentin (Bong-Soo et al., 2018; Hurley et al., 2019). Findings from recent reports were consistent with our results that revealed no clustering of microbial communities based on caries status (Jiang et al., 2016; Zhu et al., 2018; Hurley et al., 2019). Further community diversity analysis based on *S. mutans* levels showed no significant differences in alpha-diversity (Figure 4C). However, the most striking result to emerge from our data was the microbial communities significantly cluster based on *S. mutans* (HS vs. LS) (Figure 4E). The saliva from children with high *S. mutans* counts exhibited similar microbial community structure independent of their carious status, while in individuals with low *S. mutans* levels, the communities were more diverse. This result further supports the influence of *S. mutans* in shaping the composition of the microbial communities.

Intriguingly, *S. mutans* accounts for less than 1% of the community composition yet plays a crucial role in shaping the entire community by influencing the abundance of other species and shifting the oral microbiome toward disease. However, our findings highlight other disease-associated species such as *V. dispar, S. parasanguinis*, *S. intermedius*, and *Prevotella* spp., which were significantly increased in HS groups and may contribute, in combination with *S. mutans*, to the caries progression. In contrast, other health-associated species exhibited higher abundance in the LS groups, such as *V. rogosae, Haemophilus* sp., *and Alloprevotella* spp., but their contribution to the balance of health and disease is unknown.

This study is without limitations. The sample number per group could be increased to improve statistical power of the study. Although 16S rRNA gene sequencing can provide adequate phylogenetic information to identify the bacteria at the species level, for some taxa such as different *streptococcal* species, the identification is limited by the discriminatory power of the hypervariable regions and the lack of a consensus protocol. Furthermore, diet, especially the frequent intake of high carbohydrate beverages and food, is known to contribute to a sharp decrease in pH that drives a proliferation of cariogenic bacteria and microbiome dysbiosis leading to dental caries. Additional mechanistic studies should be undertaken to fully grasp the interactions between these cariogenic species and their respective contribution to caries development. The presence of different *S. mutans* strains may also account for the discordant population’s phenotypes. Previous studies demonstrated that different strains of the same species might have different contributions to caries in children (Al-Hebshi et al., 2019). Similarly, clinical isolates of *S. mutans* showed distinct phenotypic traits and thus differences in caries severity (Valdez et al., 2017). Therefore, future work should examine other potential factors contributing to the existence of the caries discordant population such as dentition stage, specific *S. mutans* strains, diet, and host susceptibility.

## Conclusion

This research has raised an important consideration of the additional species as health/disease microbial markers in conjunction with *S. mutans*. Our present unique study substantiates the understanding of the caries discordant populations microbiome and provides the first insight into how different microbes may contribute to the balance between health and disease in these populations. We believe that our research will serve as a base for future studies to unravel additional microbial targets that may help improve diagnosis and caries management of the caries discordant population.

## Data Availability Statement

The datasets presented in this study can be found in online repositories. The names of the repository/repositories and accession number(s) can be found below: ENA; PRJEB48455.

## Ethics Statement

The studies involving human participants were reviewed and approved by the University of California, Los Angeles Institutional Review Board. Written informed consent to participate in this study was provided by the participants’ legal guardian/next of kin.

## Author Contributions

MD contributed to conception, data acquisition, analysis and interpretation, and drafted and critically revised the manuscript. MA and LC contributed to data acquisition, analysis and interpretation, and drafted and critically revised the manuscript. BS drafted and critically revised the manuscript. XH, WS, DW, and RL contributed to conception, data interpretation, and critically revised the manuscript. NT contributed to conception, design, data acquisition, analysis, and interpretation, and drafted and critically revised the manuscript. All authors gave final approval and agreed to be accountable for all aspects of the work.

## Conflict of Interest

WS was a former Chief Scientific Officer of C3J Therapeutics, a biotech start-up company, where Delta Dental of Michigan is an investor. DW is a consultant to Wrigley/Mars, Colgate-Palmolive, and has equity in Liquid Diagnostics LLC. This does not alter our adherence to Frontiers Microbiology policies on sharing data and materials. This study received funding from Delta Dental. The funder was not involved in the study design, collection, analysis, interpretation of data, the writing of this article or the decision to submit it for publication. The remaining authors declare that the research was conducted in the absence of any commercial or financial relationships that could be construed as a potential conflict of interest.

## Publisher’s Note

All claims expressed in this article are solely those of the authors and do not necessarily represent those of their affiliated organizations, or those of the publisher, the editors and the reviewers. Any product that may be evaluated in this article, or claim that may be made by its manufacturer, is not guaranteed or endorsed by the publisher.

## References

[B1] Aas J. A. Griffen A. L. Dardis S. R. Lee A. M. Olsen I. Dewhirst F. E. (2008). Bacteria of dentalcaries in primary and permanent teeth in children and young adults. *J. Clin. Microbiol.* 46 1407–1417. 10.1128/JCM.01410-07 18216213 PMC2292933

[B2] Agnello M. Marques J. Cen L. Mittermuller B. Huang A. Chaichanasakul Tran N. (2017). Microbiome associated with severe caries in Canadian first nations children. *J. Dent. Res.* 96 1378–1385. 10.1177/0022034517718819 28709393 PMC5652857

[B3] Alaluusua S. Renkonen O. V. (1983). *Streptococcus mutans* establishment and dental caries experience in children from 2 to 4 years old. *Scand. J. Dent. Res.* 91 453–457. 10.1111/j.1600-0722.1983.tb00845.x 6581521

[B4] Al-Hebshi N. N. Baraniya D. Chen T. Hill J. Puri S. Tellez M. (2019). Metagenome sequencing-based strain-level and functional characterization of supragingival microbiome associated with dental caries in children. *J. Oral Microbiol.* 11:1557986. 10.1080/20002297.2018.1557986 30671194 PMC6327923

[B5] Arif N. Sheehy E. C. Do T. Beighton D. (2008). Diversity of *Veillonella* spp. from sound and carious sites in children. *J. Dent. Res.* 87 278–282. 10.1177/154405910808700308 18296614 PMC2262147

[B6] Becker M. R. Paster B. J. Leys E. J. Moeschberger M. L. Kenyon S. G. Galvin J. L. (2002). Molecular analysis of bacterial species associated with childhood caries. *J. Clin. Microbiol.* 40 1001–1009. 10.1128/JCM.40.3.1001-1009.2002 11880430 PMC120252

[B7] Belstrøm D. (2020). The salivary microbiota in health and disease. *J. Oral Microbiol.* 12:1723975. 10.1080/20002297.2020.1723975 32128039 PMC7034443

[B8] Bong-Soo K. Dong-Hun H. Ho L. Bumjo O. (2018). Association of salivary microbiota with dental caries incidence with dentine involvement after 4 years. *J. Microbiol. Biotechnol.* 28 454–464. 10.4014/jmb.1710.10028 29316741

[B9] Brailsford S. R. Tregaskis R. B. Leftwich H. S. Beighton D. (1999). The predominant *Actinomyces* spp. isolated from infected dentin of active root caries lesions. *J. Dent. Res.* 78 1525–1534. 10.1177/00220345990780090701 10512387

[B10] Caporaso J. G. Kuczynski J. Stombaugh J. Bittinger K. Bushman F. D. Costello E. K. (2010). QIIME allows analysis of high-throughput community sequencing data. *Nat. Methods* 7 335–336. 10.1038/nmeth.f.303 20383131 PMC3156573

[B11] Carlsson P. Olsson B. Bratthall D. (1985). The relationship between the bacterium *Streptococcus mutans* in the saliva and dental caries in children in Mozambique. *Arch. Oral Biol.* 30 265–268. 10.1016/0003-9969(85)90043-3 3857897

[B12] Corby P. M. Lyons-Weiler J. Bretz W. A. Hart T. C. Aas J. A. Boumenna T. (2005). Microbial risk indicators of early childhood caries. *J. Clin. Microbiol.* 43 5753–5759. 10.1128/JCM.43.11.5753-5759.2005 16272513 PMC1287835

[B13] Damle S. G. Loomba A. Dhindsa A. Loomba A. Beniwal V. (2016). Correlation between dental caries experience and mutans streptococci counts by microbial and molecular (polymerase chain reaction) assay using saliva as microbial risk indicator. *Dent. Res. J.* 13 552–559. 10.4103/1735-3327.197035 28182053 PMC5256021

[B14] Do T. Sheehy E. C. Mulli T. Hughes F. Beighton D. (2015). Transcriptomic analysis of three *Veillonella* spp. present in carious dentine and in the saliva of caries-free individuals. *Front. Cell. Infect. Microbiol.* 5:25. 10.3389/fcimb.2015.00025 25859434 PMC4374535

[B15] Dye B. A. Thornton-Evans G. Li X. Iafolla T. J. (2015). *Dental Caries and Sealant Prevalence in Children and Adolescents in the United States, 2011-2012. NCHS Data Brief.* Hyattsville, MD: U.S. Department of Health and Human Services, 1–8. 25932891

[B16] Edelstein B. L. Ureles S. D. Smaldone A. (2016). Very high salivary Streptococcus mutans predicts caries progression inyoung children. *Pediatr. Dent.* 38 325–330. 27557922

[B17] Gross E. L. Beall C. J. Kutsch S. R. Firestone N. D. Leys E. J. Griffen A. L. (2012). Beyond Streptococcus mutans: dental caries onset linked to multiple species by 16S rRNA community analysis. *PLoS One* 7:e47722. 10.1371/journal.pone.004772 23091642 PMC3472979

[B18] Gu F. Qi F. Anderson M. H. Shi W. (2006). Comparative analysis of a monoclonal antibody-based *Streptococcus mutans* detection method with selective culture assays using polymerase chain reaction as a gold standard. *Hybridoma (Larchmt)* 25 372–377. 10.1089/hyb.2006.25.372 17204000

[B19] Guo L. Shi W. (2013). Salivary biomarkers for caries risk assessment. *J. Calif. Dent. Assoc.* 41 107–118. 23505756 PMC3825179

[B20] Henne K. Rheinberg A. Melzer-Krick B. Conrads G. (2015). Aciduric microbial taxa including *Scardovia wiggsiae* and *Bifidobacterium* spp. in caries and caries free subjects. *Anaerobe* 35 60–65. 10.1016/j.anaerobe.2015.04.011 25933689

[B21] Henson B. S. Wong D. T. (2010). “Collection, storage, and processing of saliva samples for downstream molecular applications,” in *Oral Biology: Molecular Techniques and Applications*, eds Seymour G. J. Cullinan M. P. Heng N. C. K. (Totowa, NJ: Humana Press), 21–30. 10.1007/978-1-60761-820-1_2 20717775

[B22] Hurley E. Barrett M. P. J. Kinirons M. Whelton H. Ryan C. A. Stanton C. (2019). Comparison of the salivary and dentinal microbiome of children with severe-early childhood caries to the salivary microbiome of caries-free children. *BMC Oral Health* 19:13. 10.1186/s12903-018-0693-1 30642327 PMC6332856

[B23] Jiang S. Gao X. Jin L. Lo E. C. M. (2016). Salivary microbiome diversity in caries-free and caries-affected children. *Int. J. Mol. Sci.* 17:1978. 10.3390/ijms17121978 27898021 PMC5187778

[B24] Jiang W. Zhang J. Chen H. (2013). Pyrosequencing analysis of oral microbiota in children with severe early childhood dental caries. *Curr. Microbiol.* 67 537–542. 10.1007/s00284-013-0393-7 23743597

[B25] Kalpana B. Prabhu P. Bhat A. H. Senthilkumar A. Arun R. P. Asokan S. (2020). Bacterial diversity and functional analysis of severe early childhood caries and recurrence in India. *Sci. Rep.* 10:21248. 10.1038/s41598-020-78057-z 33277566 PMC7718907

[B26] Kassebaum N. J. Smith A. G. C. Bernabé E. Fleming T. D. Reynolds A. E. Vos T. (2017). Global, regional, and national prevalence, incidence, and disability-adjusted life years for oral conditions for 195 countries, 1990-2015: a systematic analysis for the global burden of diseases, injuries, and risk factors. *J. Dent. Res.* 96 380–387. 10.1177/0022034517693566 28792274 PMC5912207

[B27] Kolenbrander P. E. Jakubovics N. S. Bachrach G. (2009). “Oral microbiology,” in *Encyclopedia of Microbiology*, 3rd Edn, ed. Schaechter M. (Oxford: Academic Press), 566–588.

[B28] Loesche W. J. (1986). Role of Streptococcus mutans in human dental decay. *Microbiol. Rev.* 50 353–380. 10.1128/mr.50.4.353-380.1986 3540569 PMC373078

[B29] Ma C. Chen F. Zhang Y. Sun X. Tong P. Si Y. (2015). Comparison of oral microbial profiles between children with severe early childhood caries and caries-free children using the human oral microbe identification microarray. *PLoS One* 10:e0122075. 10.1371/journal.pone.0122075 25821962 PMC4378984

[B30] Mantzourani M. Gilbert S. C. Sulong H. N. H. Sheehy E. C. Tank S. Fenlon M. (2009). The isolation of Bifidobacteria from occlusal carious lesions in children and adults. *Caries Res.* 43 308–313. 10.1159/000222659 19494490

[B31] Marsh P. D. (1994). Microbial ecology of dental plaque and its significance in health and disease. *Adv. Dent. Res.* 8 263–271. 10.1177/08959374940080022001 7865085

[B32] Mashima I. Nakazawa F. (2014). The influence of oral Veillonella species on biofilms formed by *Streptococcus* species. *Anaerobe* 28 54–61. 10.1016/j.anaerobe.2014.05.003 24862495

[B33] Matee M. I. N. Mikx F. H. M. De Soet J. S. Maselle S. Y. De Graaff J. Van Palenstein Helderman W. H. (1993). Mutans streptococci in caries-active and caries-free infants in Tanzania. *Oral Microbiol. Immunol.* 8 322–324. 10.1111/j.1399-302x.1993.tb00582.x 8265208

[B34] Matee M. I. N. Mikx F. H. M. Maselle S. Y. M. Van Palenstein Helderman W. H. (1992). Mutans Streptococci and *Lactobacilli* in breast-fed children with rampant caries. *Caries Res.* 26 183–187. 10.1159/000261440 1628292

[B35] Nallabelli N. Patil P. P. Pal V. K. Singh N. Jain A. Patil P. B. (2016). Biochemical and genome sequence analyses of *Megasphaera* sp. strain DISK18 from dental plaque of a healthy individual reveals commensal lifestyle. *Sci. Rep.* 6:33665. 10.1038/srep33665 27651180 PMC5030485

[B36] Selwitz R. H. Ismail A. I. Pitts N. B. (2007). Dental caries. *Lancet* 369 51–59. 10.1016/S0140-6736(07)60031-2 17208642

[B37] Tanzer J. Livingston J. Thompson A. (2001). The microbiology of primary dental caries in humans. *J. Dent. Educ.* 65 1028–1037. 11699974

[B38] Valdez R. M. A. Duque C. Caiaffa K. S. Dos Santos V. R. Loesch M. L. A. Colombo N. H. (2017). Genotypic diversity and phenotypic traits of Streptococcus mutans isolates and their relation to severity of early childhood caries. *BMC Oral Health* 17:115. 10.1186/s12903-017-0406-1 28709424 PMC5512815

[B39] Wade W. G. (2013). The oral microbiome in health and disease. *Pharmacol. Res.* 69 137–143. 10.1016/j.phrs.2012.11.006 23201354

[B40] World Health Organization (1997). *Oral Health Surveys : Basic Methods*, 4th Edn. Geneva: World Health Organization.

[B41] Xu L. Chen X. Wang Y. Jiang W. Wang S. Ling Z. (2018). Dynamic alterations in salivary microbiota related to dental caries and age in preschool children with deciduous dentition: a 2-year follow-up study. *Front. Physiol.* 9:342. 10.3389/fphys.2018.00342 29670544 PMC5893825

[B42] Zhang M. Chen Y. Xie L. Li Y. Jiang H. Du M. (2015). Pyrosequencing of plaque microflora in twin children with discordant caries phenotypes. *PLoS One* 11:e0141310. 10.1371/journal.pone.0141310 26524687 PMC4629883

[B43] Zhu C. Yuan C. Ao S. Shi X. Chen F. Sun X. (2018). The predictive potentiality of salivary microbiome for the recurrence of early childhood caries. *Front. Cell. Inf. Microbiol.* 8:423. 10.3389/fcimb.2018.00423 30619773 PMC6302014

